# LIFE COURSE CONDITIONS AND COGNITION IN A NATIONALLY REPRESENTATIVE SURVEY OF OLDER ADULTS

**DOI:** 10.1093/geroni/igac059.2251

**Published:** 2022-12-20

**Authors:** James Iveniuk, Ellen Compernolle, Riddhi Gupta, Jen Hanis, Louise Hawkley

**Affiliations:** NORC at the University of Chicago, Chicago, Illinois, United States; NORC at the University of Chicago, Chicago, Illinois, United States; NORC at the University of Chicago, Chicago, Illinois, United States; NORC at the University of Chicago, Chicago, Illinois, United States; NORC at the University of Chicago, Chicago, Illinois, United States

## Abstract

By the time people reach older adulthood, their cognitive function may be conserved, or in decline, in part due to their social experiences over their entire life course. Researchers have gained a greater understanding, over recent decades, of the importance of life-course events for cognition in later life. Nevertheless, our understanding of many of these factors, especially in childhood, remains limited. Drawing upon Round 2 of the National Social life, Health, and Aging Project (NSHAP; N=3,377), and data linked to the 2010 census, the 1940s census, and air pollution data, we undertake a whole-life-course approach to understanding the determinants of cognitive function in older adults. Building on the work of the Lancet Commission on risk factors for dementia, we considered health conditions, low education,incarceration, and brain injury (ever); poor health behaviors and low social contact (current); and air pollution (average over past five years). We also considered adverse childhood experiences,, and home conditions in 1940. Similar to other studies, we found that female gender, identifying as white, and being born in the US were significantly associated with better cognitive function. Higher depression and lower social contact were associated with worse cognition. There were no significant associations between cognition and early childhood factors - with the exception that growing up in an urban area was associated with better cognitive function. Experiencing jail time was also negatively associated with cognitive function. Findings point towards the need for a more expansive consideration of life course conditions, as they impact cognition in late life.

